# Influence of kinesiophobia on activity, function, and anxiety levels in patients with rheumatoid arthritis

**DOI:** 10.3389/fmed.2024.1514088

**Published:** 2025-01-15

**Authors:** Shang Xueying, You Yanli, Xu Wei, Zhang Lingling, Li Lili

**Affiliations:** ^1^Department of Critical Care Surgery, The First Affiliated Hospital of Henan University of Science and Technology, Luoyang, China; ^2^School of Nursing, The First Affiliated Hospital of Henan University of Science and Technology, Luoyang, China; ^3^Department of Gastrointestinal Surgery, The First Affiliated Hospital of Henan University of Science and Technology, Luoyang, China; ^4^Health Management Center, The First Affiliated Hospital of Henan University of Science and Technology, Luoyang, China

**Keywords:** physical activity, kinesiophobia, pain-related anxiety, rheumatoid arthritis, anxiety

## Abstract

**Background:**

Rheumatoid arthritis (RA) is a chronic autoimmune disorder that causes joint inflammation and affects quality of life. Appropriate physical activity can enhance joint function and lower cardiovascular disease risk. However, individuals with RA often have reduced physical activity levels, likely due to kinesiophobia, or fear of movement.

**Aim:**

This study aimed to assess the prevalence of kinesiophobia among RA patients and its influence on functional impairment, physical activity, and pain-related anxiety.

**Methods:**

Using a convenience sampling method, we surveyed 350 RA patients attending outpatient clinics in the rheumatology and immunology departments of three tertiary hospitals in Henan Province, China, from August 18 to September 1, 2023. Participants completed the Tampa Scale of Kinesiophobia (TSK), the Signals of Functional Impairment Scale (SOFI), the International Physical Activity Scale—Short Form (IPAQ-SF), and the Pain Anxiety Symptoms Scale-20 (PASS-20). The Disease Activity Score 28 (DAS28) was retrieved for each participant to assess disease activity in RA patients. Descriptive analysis, Chi-square tests, Spearman correlation, and multiple linear regression assessed factors influencing kinesiophobia, with significance set at *p* < 0.05.

**Results:**

Results indicated that 70.86% of participants experienced kinesiophobia, which was positively correlated with functional impairment and pain-related anxiety, while inversely related to physical activity levels (*p* < 0.001). Regression analysis revealed that kinesiophobia was explained by 65.5% of the variance, with gender, education level, functional impairment, pain-related anxiety, and pain severity identified as significant predictors (*p* < 0.05).

**Conclusion:**

The findings suggest that RA patients exhibit a high prevalence of kinesiophobia, predominantly influenced by factors such as gender, lower educational attainment, increased pain levels, greater functional impairment, and pain-related anxiety. Notably, physical activity levels did not serve as a predictor of kinesiophobia in this cohort.

## 1 Introduction

Rheumatoid arthritis (RA) is an autoimmune disorder characterized by inflammation of the synovial joints (1). This progressive and chronic condition manifests with symptoms such as pain, swelling, morning stiffness, and restricted joint mobility (2). Over time, the disease can lead to joint deformities and functional limitations, as well as damage to vital organs, including the heart and lungs (1, 2). Consequently, RA significantly affects the quality of life (QoL) of those affected. Epidemiological studies indicate that the global incidence of RA ranges from 0.5 to 1.0%, with a prevalence of 0.28 to 0.45% in China (3).

The primary objective of RA management is to control disease activity through early intervention, coupled with ongoing follow-up and monitoring. Effective management aims to delay disease progression, prevent joint destruction, reduce disability rates, and enhance the physical, psychological, and social well-being of patients (4). Physical activity, recognized as a non-pharmacological intervention, has been demonstrated to alleviate systemic symptoms and decrease healthcare costs for RA patients (5). Evidence suggests that increased physical activity can lower pain levels, improve muscle strength and aerobic capacity, maintain or enhance joint function, and reduce fatigue and cardiovascular disease risk, thereby improving overall QoL (4, 5).

Despite the known benefits of physical activity, many RA patients exhibit lower activity levels compared to the general population (6–8). This phenomenon may be associated with kinesiophobia, defined as an excessive and irrational fear of movement or physical activity, often stemming from heightened pain sensitivity and prior injuries (9–11). Chronic joint pain experienced by RA patients can result in fear of reinjury, leading to kinesiophobia (10, 12). This fear can further contribute to decreased activity, heightened pain, and increased risk of limb dysfunction and disability, along with elevated levels of depression and anxiety, adversely impacting QoL (13).

Studies on kinesiophobia in RA patients remain limited. In particular, studies have reported a high incidence of kinesiophobia, which adversely affects pain levels, disease activity, functional status, and psychological well-being (14, 15). In addition, pain-related anxiety (characterized by cognitive, emotional, behavioral, and physiological responses to pain) has been linked to fear-avoidance behaviors (16–18). However, the relationship between kinesiophobia, functional impairment, physical activity levels, and pain-related anxiety in RA patients requires further investigation. This study aims to explore kinesiophobia in RA patients and its relationship with functional impairment, physical activity, and pain-related anxiety symptoms.

## 2 Materials and methods

### 2.1 Ethical aspects

This study was approved by the Ethics Committee of the First Affiliated Hospital of Henan University of Science and Technology (permission number: 2023–687), following the Declaration of Helsinki. All protocols followed the relevant guidelines and regulations, and informed consent was obtained from all subjects involved in the study.

### 2.2 Study participants

We conducted a cross-sectional study to assess kinesiophobia in patients with RA and its relationship with the degree of functional impairment, physical activity level, and pain-related anxiety. The study was carried out from August 18th to September 1st, 2023, and included RA patients who attended the outpatient clinics of rheumatology and immunology at three tertiary hospitals in Henan Province, China. Data were collected using questionnaires and interviews and analyzed using descriptive statistics, Spearman correlation, and regression analysis.

The inclusion criteria were (1) participants meeting the RA diagnostic criteria jointly developed by the American Rheumatism Association (ACR) and the European League Against Rheumatism (EULAR) in 2010; (2) age ≥ 18 years old; (3) those with certain reading comprehension and language expression skills; (4) those who participated voluntarily and provided written informed consent. Exclusion criteria included: (1) those with impaired consciousness or accompanied by hearing impairment; (2) those with a history of previous mental illness and/or family mental illness; (3) participants with heart, lung, brain, and other important organ damage; (4) combined with other rheumatic immune diseases; (5) those who underwent surgery and were in unstable condition; (6) potential participants with cardiovascular and cerebrovascular diseases, chronic obstructive pulmonary disease, malignant tumors, venous thrombosis of the lower extremities, and other diseases that affect exercise.

### 2.3 Sample size

Recent studies confirmed that the incidence of kinesiophobia in RA patients is about 70% (14). Bilateral tests were required to *α* test at 0.05, the power of test 1-*β* was 0.8, and the allowable error was 0.05. PASS15 (NCSS LLC, Kaysville, USA) was used to calculate the required sample size, which was 323 cases. Considering that 10% of the questionnaires may be invalid, the final sample size was 359 cases.

### 2.4 Scales and questionnaires

The following scales and questionnaires were used in this study:

(a) General information questionnaire: This was self-compiled and included information on the age, sex, marital status, place of residence, educational level, occupational status, Body Mass Index (BMI), disease activity, medication, and pain level of the patient.
(b) Tampa Scale of Kinesiophobia (TSK): This scale was created by Woby et al. and is primarily used to assess anxiety associated with movement in patients experiencing chronic pain (19–21). The scale after sinicization included 3 dimensions and 11 items, using a Likert 4-level method of scoring with a total score range of 11–44 points. Higher scores indicate higher levels of kinesiophobia, with scores >26 points defined as kinesiophobia. The Cronbach’s alpha is 0.883, with a reliability of retest of 0.798 (21, 22). In the present study, the TSK had a Cronbach’s alpha of 0.863.
(c) Signals of Functional Impairment (SOFI): This scale is mainly used to assess the degree of limb joint dysfunction and includes three sections that specifically assess hand, upper limb, and lower limb function. Each part consists of 4 items, with a scoring of 0 for normal, 1 for partial impairment, and 2 for inability to perform. The scores for each part of the bilateral limb score are between 0 and 16, with the total score ranging between 0 and 48 points. Higher scores indicate higher levels of impairment (15). The reliability correlation coefficient of the retest was >0.750, and the reliability, validity, and sensitivity were good (15). Here, the SOFI had a Cronbach’s alpha coefficient of 0.956.
(d) International Physical Activity Questionnaire (IPAQ-SF; SF, short form): This scale was jointly developed by experts from the International Physical Activity Group in 2001 (23). In 2004, it was translated to Chinese and was found to have good reliability and validity (24). This scale consists of 7 items and is mostly used as an assessment of a person’s physical activity over the previous 7 days, including low-intensity (MET assignment 3.3), moderate-intensity (MET assignment 4.0), and high-intensity (MET assignment 8.0) activity. The standard duration for any activity is a minimum of 10 min (24, 25). The assessment of individual activity levels is calculated as the MET assignment of the intensity * weekly frequency (d/w) * daily time (min/d), with the sum of the values for the three activity levels representing the total physical activity. The overall activity is graded, with any one of the following considered to represent high-level activity: (1) a minimum of 3 days with high-intensity activity with a weekly total of ≥1,500 MET-min/w; (2) a minimum of 7 days including activity at each level of intensity, with a weekly total of 3,000 MET-min/w. (2) Medium activity is defined as: (1) at least 20 min of high-intensity activity per day over ≥3 days; (2) at least 30 min of any type of moderate-intensity and/or walking activities per day for ≥5 days; (3) five days with physical activity of any level, with an overall value of ≥600 MET-min/w per week. Low-level activity is defined as no activity or activity at a level below those defined by high and medium activity.
(e) Pain-related anxiety symptoms scale-20 (PASS-20): The scale was created by McCracken et al. and is primarily used to assess pain-related anxiety (26). Zhou et al. sinicized the scale, which showed good reliability and internal consistency, with a Cronbach alpha of 0.92 and an intraclass correlation coefficient (ICC) of 0.90 (18). PASS-20 contains 4 dimensions, namely, avoidance behavior, cognitive anxiety, fear of pain, and physiological symptoms, with a total of 20 items. Using a 6-scale scoring system, from 0 (never) to 5 (always), the total score is 100 points; 0–34 points represent mild pain-related anxiety, 35–67 points represent moderate pain-related anxiety; 68–100 points indicate severe pain-related anxiety. In this study, the PASS-20 scale had a Cronbach’s coefficient of 0.975.

### 2.5 Procedure

The purpose of the study was explained to participants in person before the study began, and their informed consent was obtained. Subsequently, the researcher completed the Signals of Functional Impairment scale for the respondent, while the other scales were completed by the respondent under the guidance of the researcher. The completed questionnaires were collected and inspected by the investigators to avoid invalidity in the questionnaires.

### 2.6 Statistical analysis

Data were entered and analyzed using SPSS v. 26.0 (IBM, Armonk, United States). Data were entered independently and cross-checked by two investigators. A descriptive analysis of data was performed. Chi-square tests were used in univariate analysis. Spearman correlation analysis was conducted and factors influencing kinesiophobia were assessed by multiple linear regression. Regression analysis was used to assess variance homogeneity of the residuals using standardized distributions and scatterplots. Residual normality was examined by analyzing the normalized residual histogram and residual independence using the Durbin–Watson statistic. *p*-values <0.05 were considered statistically significant.

## 3 Results

### 3.1 Demographic data of participants

A total of 359 questionnaires were distributed and 350 valid questionnaires were recovered (97.5%). The 350 RA patients included 276 women (78.9%); 321/350 were married (91.7%); The age of the participants was 56.28 ± 0.64 years with RA for 9.07 ± 0.52 years (Table 1). In addition, 191 (54.6%) patients lived in city/town areas and 159 (45.4%) lived in rural areas; There were 106 (30.3%) patients with primary/or no formal school education, 121 (34.6%) were trained up to junior Chinese students, 73 (20.9%) with senior Chinese, and 50 (14.2%) with a college diploma or above. 64 (18.3%) were working, 104 (29.7%) were retired, and 182 (52.0%) belonged to others. According to DAS28, there were 71 (20.3%) patients in clinical remission, 65 (18.6%) in the low disease activity phase, 152 (43.4%) in the medium disease activity phase, and 62 (17.7%) in the high disease activity phase. In this study, the total TSK-11 score was 29.29 ± 0.33, and the incidence of kinesiophobia was 70.86% (248/350).

**Table 1:** Comparison of social demographic factors between RA patients with and without kinesiophobia (*N* = 350).

Variable	With kinesiophobia (*N* = 248)	Without kinesiophobia (*N* = 102)		Total	*c* ^2^	*p*
Age (years)					0.544	0.762
18–44	43 (17.4)	17 (16.7)		60 (17.1)		
45–59	106 (42.7)	40 (39.2)		146 (41.7)		
≥60	99 (39.9)	45 (44.1)		144 (41.2)		
Gender					25.224	<0.001
Male	35 (14.1)	39 (38.2)		74 (21.1)		
Female	213 (85.9)	63 (61.8)		276 (78.9)		
Residence					3.005	0.083
City/Town	128 (51.6)	63 (61.8)		191 (54.6)		
Rural	120 (48.4)	39 (38.2)		159 (45.4)		
Marital status					0.384	0.536
Married	226 (91.1)	95 (93.1)		321 (91.7)		
Single or separated	22 (8.9)	7 (6.9)		29 (8.3)		
Educational levels					58.725	<0.001
Primary/or no formal school education	97 (39.1)	9 (8.8)		106 (30.3)		
Junior school	92 (37.1)	29 (28.4)		121 (34.6)		
Senior school	40 (16.1)	33 (32.4)		73 (20.9)		
College or above	19 (7.7)	31 (30.4)		50 (14.2)		
Work status					1.710	0.425
Working	45 (18.2)	19 (18.6)		64 (18.3)		
Retired	69 (27.8)	35 (34.3)		104 (29.7)		
Other	134 (54.0)	48 (47.1)		182 (52.0)		
BMI (kg/m^2^)					5.103	0.164
BMI<18.5	28 (11.3)	9 (8.8)		37 (10.6)		
18.5 ≤ BMI<24	138 (55.6)	51 (50.0)		189 (54.0)		
24 ≤ BMI<28	62 (25.0)	37 (36.3)		99 (28.3)		
BMI ≥ 28	20 (8.1)	5 (4.9)		25 (7.1)		
DAS28					24.765	<0.001
Remission (DAS28<2.6)	34 (13.7)	37 (36.3)		71 (20.3)		
Low disease activity (2.6 ≤ DAS28<3.2)	49 (19.7)	16 (15.7)		65 (18.6)		
Moderate disease activity (3.2 ≤ DAS28 ≤ 5.1)	113 (45.6)	39 (38.2)		152 (43.4)		
High disease activity (DAS28>5.1)	52 (21.0)	10 (9.8)		62 (17.7)		
Disease duration (years)					2.861	0.581
<1.0	19 (7.7)	10 (9.8)		29 (8.3)		
1.0–5.0	97 (39.1)	45 (44.1)		142 (40.6)		
5.1–10.0	53 (21.4)	22 (21.6)		75 (21.4)		
10.1–15.0	30 (12.1)	12 (11.8)		42 (12.0)		
>15.0	49 (19.7)	13 (12.7)		62 (17.7)		
Pain duration (months)					1.537	0.464
<1	158 (63.7)	71 (69.6)		229 (65.4)		
1–3	72 (29.0)		23 (22.6)	95 (27.2)		
≥3	18 (7.3)	8 (7.8)		26 (7.4)		
Type of medication					3.293	0.349
1 type	56 (22.6)	19 (18.6)		75 (21.4)		
2 type	103 (41.5)	50 (49.0)		153 (43.7)		
3 type	74 (29.8)	24 (23.6)		98 (28.0)		
4 type and above	15 (6.1)	9 (8.8)		24 (6.9)		
Co-morbidities						

BMI, Body mass index; DAS28, Disease activity score in 28 joints.

### 3.2 Univariate analysis of kinesiophobia in RA patients

Women had higher levels of kinesiophobia than men (*c*^2^ = 25.224, *p* < 0.001). In addition, the levels of kinesiophobia were found to correlate with the Disease Activity Score in 28 Joints (*c*^2^ = 24.765, *p* < 0.001). A correlation between kinesiophobia and educational level was also observed, with patients with high formal literacy levels having lower levels of kinesiophobia (*c*^2^ = 58.725, *p* < 0.001) (Table 2).

**Table 2:** Spearman correlations between the study variables.

*N* = 350	Spearman correlation	*p*
VAS	0.588	<0.001
DAS28	0.427	<0.001
SOFI total	0.556	<0.001
PASS total	0.602	<0.001
IPAQ total	−0.253	<0.001

VAS, visual analog scale; DAS28, Disease activity score in 28 joints; SOFI, Signals of Functional Impairment; PASS, pain-related anxiety symptoms scale; IPAQ, International Physical Activity Questionnaire.

### 3.3 Spearman correlations between the study variables

TSK-11 was positively correlated with pain levels (*r* = 0.588), indicating that high levels of kinesiophobia were strongly correlated with strong levels of pain. TSK was also correlated with DAS28, functional impairment, and pain-related anxiety, with r values of 0.427, 0.556, and 0.602, respectively (*p* < 0.001) (Table 2). However, physical activity and kinesiophobia were negatively associated, with higher activity levels correlated with lower kinesiophobia, but the correlation was weak (*r* = −0.253, *p* < 0.001).

### 3.4 Multivariate linear regression analysis of kinesiophobia associations

Multiple linear regression analysis was performed with TSK11 scores as a dependent variable, and gender, education level, DAS28 score, PASS, SOFI, VAS score, and IPAQ level as independent variables. Multicollinearity diagnosis was conducted by determination of the variance inflation factor (VIF) and tolerance. Since all VIFs were below 3 and the tolerance was above 0.4, we confirmed the absence of multicollinearity. In addition, the multiple linear regression model explained 65.5% of the factors affecting kinesiophobia (*R*^2^ = 0.655, *p* < 0.001) (Table 3).

**Table 3:** Multivariate linear regression analysis of kinesiophobia associations.

					95%CI	
Variable		B	β	*p*	Lower	Upper
Gender						
	Male (reference)					
	Female	2.158	0.144	<0.001	1.170	3.146
Literacy						
	Primary school and illiteracy (reference)					
	Junior high school	−1.101	−0.086	0.031	−2.103	−0.099
	Senior middle school	−2.103	−0.140	0.001	−3.304	−0.901
	College degree or above	−3.925	−0.224	<0.001	−5.257	−2.592
SOFI		0.218	0.370	<0.001	0.173	0.263
PASS		0.053	0.206	<0.001	0.032	0.073
DAS28		−0.167	−0.041	0.332	−0.506	0.171
VAS		0.533	0.263	<0.001	0.350	0.715
IPAQ level						
	Low (reference)					
	Medium	0.419	0.028	0.389	−0.537	1.375
	High	−0.293	−0.014	0.670	−1.646	1.059

SOFI, Signals of Functional Impairment; PASS, pain-related anxiety symptoms scale; IPAQ, International Physical Activity Questionnaire. DAS28, Disease activity score in 28 joints; VAS, visual analog scale.

## 4 Discussion

Patients with rheumatoid arthritis (RA) frequently experience long-term joint pain, which can lead to the development of an excessive and irrational fear of physical activity and rehabilitation exercises, resulting in avoidance behaviors (1, 2). In this study, the incidence of kinesiophobia among participants was 70.9%, aligning with previous findings of 70% (14), while being higher than that observed in patients with multiple sclerosis (61.2%) (27) and lower than in individuals with knee osteoarthritis (85.7%) (28). Several factors may account for these variations.

First, kinesiophobia can manifest across various diseases, with its prevalence influenced by disease type, clinical manifestations, and demographic characteristics of the study population. Differences in disease, geographic region, and cultural context can significantly affect incidence rates (14, 15). For this reason, future studies should consider multi-center, cross-regional, large-sample cross-sectional studies to provide a comprehensive understanding.

Second, the existence of numerous assessment tools for kinesiophobia may lead to inconsistent findings due to variations in methodology employed by different investigators (2, 21). Each tool may have different constructs, measurement scales, and validation processes, which can lead to discrepancies in the prevalence and severity of kinesiophobia reported in the literature. Some tools may focus on specific dimensions of fear related to movement, while others might assess broader psychological factors, leading to variations in outcomes. To address these inconsistencies, it is essential to standardize assessment methods for kinesiophobia, ensuring that all tools are rigorously validated and reflect the multifaceted nature of the condition. Establishing a consensus on the most effective assessment instruments could enhance the reliability of results and facilitate more accurate comparisons across studies.

Third, the scientific literature on kinesiophobia in RA remains limited and primarily focuses on quantitative and cross-sectional studies, which limits the applicability of findings. Consequently, qualitative and longitudinal studies are essential to explore the influencing factors of kinesiophobia in RA patients, providing valuable insights for clinical interventions.

Lastly, clinical practitioners often overlook kinesiophobia in RA patients, leading to inadequate management and awareness of this issue (29). Increased dissemination of information regarding kinesiophobia is necessary, along with enhancing assessment, diagnosis, and treatment strategies to correct patients’ misconceptions and develop effective self-management techniques.

The results of our study indicated that female RA patients presented a higher likelihood of experiencing kinesiophobia compared to their male counterparts, consistent with findings from a Turkish study (14) and supported by observations in a Norwegian study that reported higher kinesiophobia levels in women with chronic low-back pain (30). This tendency may be attributed to women’s heightened pain sensitivity and lower psychological tolerance for pain (31–33). Furthermore, the analysis revealed that lower literacy levels were associated with increased kinesiophobia, corroborating multiple studies (15, 18). Patients with limited literacy may have reduced access to disease-related information, leading to misconceptions about exercise and its role in pain relief. They are also more prone to cognitive errors, further exacerbating their fears (34). A positive correlation between pain levels and the degree of kinesiophobia was also observed, reflecting the findings from Asiri et al. and Yildiz et al. (35, 36). The fear-avoidance model suggests that persistent pain can lead to catastrophic thinking about pain, resulting in reduced movement and a cycle of increased pain sensitivity and misperception (35, 36). This highlights the necessity for healthcare providers to focus on women and patients with lower educational attainment, addressing kinesiophobia through timely intervention and education.

Our study also identified that functional impairment in RA patients was both associated with and predictive of kinesiophobia, corroborating Baday-Keskin and Ekinci (15). They posited that disability may exacerbate kinesiophobia, worsening existing dysfunction. This relationship has been observed in other studies, indicating that RA patients require not only pharmacological management but also rehabilitation exercises to recover or maintain limb function (37). However, those experiencing kinesiophobia often harbor strong fear-avoidance beliefs, which can lead to further dysfunction (38, 39). Therefore, assessing kinesiophobia is essential for correcting misconceptions and enhancing limb function. Clinical staff should implement psychological interventions to reduce fear-avoidance beliefs, encouraging participation in functional exercises to improve quality of life. Establishing a multidisciplinary rehabilitation team could also assist patients in understanding the benefits and methods of exercise.

While the study demonstrated a correlation between kinesiophobia and physical activity levels, it did not predict the occurrence of kinesiophobia. Low physical activity levels in RA patients may arise from various complex factors, including disease symptoms, psychological disorders, and environmental influences (6–8). Although some studies have noted a negative correlation between kinesiophobia and physical activity levels (7, 8), others have not observed such correlation (15, 40). These inconsistencies may stem from differences in clinical symptoms among patient populations and the tools employed to measure physical activity. In this study, RA patients exhibiting kinesiophobia demonstrated lower physical activity levels than those without, emphasizing the need for strategies to reduce sedentary behavior and improve activity levels to decrease risks of cardiovascular disease and dysfunction. Future investigations should focus on identifying suitable tools for accurately assessing physical activity in RA patients.

Our study also revealed a significant correlation between pain-related anxiety and high levels of kinesiophobia in RA patients, with pain-related anxiety serving as a predictor of kinesiophobia. Mehta et al. demonstrated an association between activity avoidance and adverse mood in RA patients, who are susceptible to depression and anxiety due to chronic pain (41). These emotional disorders can exacerbate avoidance behaviors and worsen pain levels and disease activity (42, 43). Jiang et al. also noted a moderate correlation between pain-related anxiety and kinesiophobia (43). According to the fear-anxiety-avoidance model, pain-related anxiety plays a significant role in developing fear-avoidance behaviors (17). Joint pain in RA patients often intensifies during physical activity, leading to anxiety about exacerbating pain and disease, which fosters avoidance and ultimately results in kinesiophobia. The relationship between pain-related anxiety and kinesiophobia necessitates that healthcare providers monitor emotional changes in patients and employ appropriate psychological interventions to alleviate negative emotions and prevent kinesiophobia.

While this study contributes to the understanding of kinesiophobia in RA, it also has some limitations. The cross-sectional design of this study precludes the establishment of causal relationships between kinesiophobia and the outcomes of interest. While correlations can be observed, it is not possible to determine the direction of influence or rule out the impact of confounding variables. Longitudinal studies are necessary to investigate the causal pathways and temporal relationships between kinesiophobia and patient outcomes. In addition, the use of convenience sampling may affect the homogeneity of the patient population, potentially limiting the generalizability of the findings. Future studies should aim to employ longitudinal designs and diverse sampling methods to further elucidate the mechanisms underlying kinesiophobia in RA patients and develop targeted interventions to address this critical issue. Finally, the reliance on self-reported measures of kinesiophobia and related constructs introduces the potential for recall bias and social desirability bias. Participants may not accurately recall their experiences or may respond in ways they perceive as more socially acceptable, potentially affecting the validity of the findings. Future research could incorporate objective measures of kinesiophobia, such as physiological responses to movement-related stimuli, to mitigate these biases.

## 5 Conclusion

Kinesiophobia is prevalent among patients with RA, with functional impairment and pain-related anxiety serving as significant predictors of its severity. However, physical activity levels do not significantly predict the fear of movement in these patients. It is essential for clinical medical staff to remain vigilant regarding the presence and contributing factors of kinesiophobia in RA patients. Enhancing daily screening and assessment protocols can facilitate the identification of effective management strategies aimed at reducing patients’ fears related to movement. Furthermore, the establishment of a comprehensive multidisciplinary clinical team may provide valuable support, enabling patients with RA to improve both their physical and mental well-being.

## Data Availability

The original contributions presented in the study are included in the article/supplementary material, further inquiries can be directed to the corresponding author.
